# Diagnosis of Enlarged Vestibular Aqueduct Using Wideband Tympanometry

**DOI:** 10.3390/jcm13216602

**Published:** 2024-11-03

**Authors:** Akira Ganaha, Nao Nojiri, Takeshi Nakamura, Teruyuki Higa, Shunsuke Kondo, Tetsuya Tono

**Affiliations:** 1 Department of Otorhinolaryngology, Head and Neck Surgery, International University of Health and Welfare, Narita Hospital, Narita 286-8520, Japan; nao-nojiri@iuhw.ac.jp; 2 Department of Otolaryngology and Head and Neck Surgery, Faculty of Medicine, University of Miyazaki, Miyazaki 889-1692, Japan; takeshi_nakamura@med.miyazaki-u.ac.jp; 3 Department of Otorhinolaryngology-Head and Neck Surgery, University of the Ryukyus, Nishihara 903-0215, Japan; tellurteru@yahoo.co.jp (T.H.); kouhouiinn@yahoo.co.jp (S.K.); 4 Department of Otolaryngology, International University of Health and Welfare, Tochigi 324-8501, Japan; tono@med.miyazaki-u.ac.jp

**Keywords:** wideband tympanometry, enlarged vestibular aqueduct, diagnosis, ROC analysis, absorbance, resonance frequency, temporal bone computed tomography

## Abstract

**Background:** Wideband tympanometry (WBT) has the potential to distinguish various mechanical middle ear and inner ear pathologies noninvasively. This study investigated the diagnostic value of WBT in the diagnosis of enlarged vestibular aqueduct (EVA). **Methods:** The absorbance and resonance frequency (RF) of patients with EVA (40 ears, 25 patients) and matched population controls (39 ears, 28 subjects) were compared, alongside receiver operating characteristic (ROC) analysis. Correlations between VA width and RF were also examined. **Results:** Patients with EVA had higher absorbance at low frequencies (226–917 Hz) and lower absorbance at high frequencies (2520–4896 Hz) compared to controls. The RF of the EVA group was significantly lower versus controls (751 [391–1165] vs. 933 [628–1346] Hz). The ROC analysis revealed area under the curve values of 0.771 and 0.801, respectively, for absorbance and RF. RF had a sensitivity, specificity, positive predictive value, and negative predictive value of 74.4%, 82.5%, 76.7%, and 80.6%, respectively, for diagnosing EVA. In the EVA group, the VA midpoint width (r = −0.334) and VA petrous width (r = −0.402) both significantly correlated with RF. **Conclusions:** Our findings support the utility of WBT for diagnosing EVA, with RF as the optimal index used.

## 1. Introduction

Tympanometry using low tone stimulation is widely used as a noninvasive, simple, and inexpensive instrument for diagnosing middle ear disease [1]. Due to its high sensitivity, the usefulness of tympanometry using high-pitched sounds has become clinically recognized [1]. Wideband tympanometry (WBT) has a wider stimulation frequency range (226–8000 Hz) and can provide more detailed information about the condition of the middle and inner ear in terms of acoustic energy absorption at different frequencies and different pressures. The results of WBT measurements are expressed as wideband reflectance (WBR) or wideband absorbance (WBA) [2]. While WBR is defined as the ratio of the energy incident on the ear canal to the energy reflected from the eardrum, WBA represents the part of the incident energy that is absorbed by the middle ear. Both WBA and WBR range from 0 to 1, with WBA being equal to 1-WBR. Another parameter evaluated is resonance frequency (RF), which is the frequency at which the mass and stiffness effects of the middle ear are equal. The sound at RF is transmitted more readily by the middle ear than sounds at other frequencies [3]. Thus, both WBA and RF are measured, which are different from those obtained via tympanometry, providing an extensive insight into the middle and inner ears and facilitating the diagnosis of various pathologies [4].

The pressure of the cerebrospinal fluid is transmitted to the endolymph through the endolymphatic sac, and to the perilymph through the cochlear aqueduct [5]. Thus, when the cerebrospinal fluid pressure increases, both the endolymphatic and perilymphatic pressures increase accordingly. An increase in cerebrospinal fluid pressure causes an increase in cochlear pressure, which in turn increases acoustic impedance in the tympanic membrane and the middle ear conductive system [6,7]. Furthermore, an experimental report by Murakami et al. has shown that an increase in inner ear pressure causes a decrease in stapes vibration and tympanic membrane vibration [6]. Thus, the noninvasive measurement of middle ear transfer function using WBT can be used to determine the condition of the inner ear indirectly. The recent emergence of WBT has demonstrated its effectivity in diagnosing inner ear diseases that affect inner ear pressure, such as superior semicircular canal dehiscence syndrome (SSCD) [4] and Meniere’s disease [8].

An enlarged vestibular aqueduct (EVA) is the most common inner ear anomaly causing congenital sensorineural hearing loss, which is characterized by an air–bone gap in low frequencies with fluctuating and progressive hearing loss [9,10]. There is increased perilymphatic pressure during cochlear implantation, and a negative summating potential suggests the presence of endolymphatic hydrops [11,12]. This may push the stapes up from the inside and restrict its movement [13]. Such anatomical abnormalities that allow communication between the inner ear fluid space and the cranial cavity are called third-window lesions, and similar disorders such as SSCD have been reported. Thus, increased inner ear pressure also affects the middle ear conductive system. The early diagnosis of EVA is an important clinical challenge. Current diagnostic strategies rely on expensive imaging; thus, the interventions are usually delayed. There is an urgent need for simpler, cost-effective diagnostic tools, as EVA accounts for 7%–13% of all cases of congenital hearing loss [14]. Although WBT offers potential advantages in terms of noninvasiveness, rapid testing, and decreased cost, it has never been adequately explored for its diagnostic precision pertaining to EVA. Previous research has revealed encouraging results of WBT on other inner ear pathologies, suggesting its potential application in the diagnosis of EVA [4,8,15,16]. In this report, we compared patients with EVA with a distribution-matched control group to clarify the characteristics of WBT in patients with EVA and evaluate the usefulness of WBT in diagnosing EVA.

## 2. Materials and Methods

### 2.1. Subjects

WBT was performed from June 2019 to April 2022 for patients with EVA (EVA group) and normal control subjects (control group) in the Department of Otorhinolaryngology, Head and Neck Surgery of the University of the Ryukyus, Japan, and in the Department of Otorhinolaryngology, Head and Neck Surgery of Miyazaki University, Japan.

The inclusion criteria for both groups are described in Table 1. The inclusion criteria for the control group were as follows: (1) no history of ear diseases; (2) no acute or chronic upper respiratory inflammation; (3) normal findings on pretest otoscopic examination using oto-microscopy; (4) normal type-A 226 Hz tympanometry (peak pressure between 50 and +50 daPa with a single peak with the static admittance values of 0.3–1.7 mmho) [17]; (5) normal audiometric thresholds (≤25 dBHL from 500 to 2000 Hz; air–bone gap ≤10 dB from 500 to 2000 Hz); and (6) no allergic rhinitis, enlarged tonsils, adenoid hyperplasia, or acute or chronic upper respiratory disease. The inclusion criteria for the EVA group were (1) meeting inclusion criteria (1) to (5) in the control group as shown earlier; (2) a radiological diagnosis of EVA (i.e., VA with a diameter of >1.5 mm at the midpoint); and (3) no history of middle or inner ear surgery (e.g., tympanostomy tube, tympanoplasty, or cochlear implant surgery). A power analysis was conducted based on preliminary data from a pilot study in 20 subjects (10 EVA patients and 10 controls); a sample size of 46 participants (23 EVA patients and 23 controls) was determined to yield 80% power, facilitating the detection of a clinically meaningful difference of 0.05 in RF between EVA and control groups. This study was conducted with approval from the ethics committee of the University of Miyazaki (registration number: O-0617) and the University of the Ryukyus (registration number: 1582).

### 2.2. Image Evaluation

EVA was diagnosed via temporal bone computed tomography (CT) in all patients, specifically ultrahigh-resolution CT (Aquilion PrecisionVR; Canon Medical Systems, Tochigi, Japan) with a 0.25 mm slice thickness or high-resolution CT (Aquilion One; Canon Medical Systems, Tochigi, Japan) with a 0.5 mm slice thickness. EVA was defined as a VA with a diameter of >1.5 mm at the midpoint between the common crus of the semicircular canal and the external aperture of the VA on CT [18].

**Figure 1:**
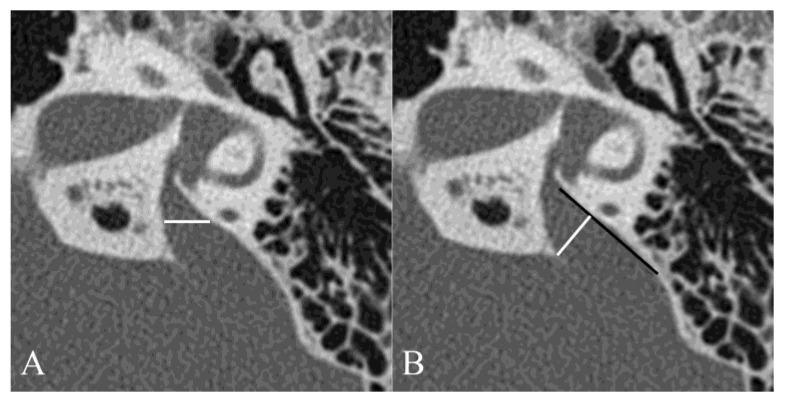
Measuring the VA midpoint and porous width in axial computed tomography images. The VA midpoint width (white line) was measured at the half the distance from VA fundus to its external pore (**A**). The VA porous width (white line) was measured from the opercular margins to the spots on the posterior temporal bone walls (black line) whose surface was perpendicular to the measurement lines (**B**).

The VA width was measured using two different methods (Figure 1) [19]. The VA midpoint width was defined as the part of VA in the petrous bone located half the distance from its origin in the labyrinth to its aperture in the epidural space (Figure 1A). The VA opercular width was measured in the same plane as the endolymph sac depths by drawing a line from the opercular edge anterolaterally to form a 90° angle with the posterior wall of the petrous bone (Figure 1B) [19,20]. VA was measured by two neuro-otologists (GA and NN). Interobserver variability at the two measurement points was investigated.

### 2.3. Measurement of WBT

The Titan^®^ (IMP440 version 3.4, Interacoustics, Middelfart, Denmark) was used to measure the power absorbed in the ear canal (frequency range, 226–8000 Hz; pressure sweep, −300–+200 daPa; rate, 100 daPa/s). Power absorbance was defined as a ratio of absorbed power over the incident power, ranging from 0 to 1. A power absorbance of 0 and 1, respectively, means that all sound energy has been reflected back or absorbed by the middle ear system [21]. A probe validity check was performed using the built-in 2 cc cavity of the Titan cradle. A suitable ear tip was carefully selected to ensure a proper fit and good seal of the ear canal. WBT measurements were performed in triplicate according to a unified protocol, including equipment calibration every 6 months and soundproofing, to ensure reliability. Quality control measures were included. Intraclass correlation coefficients were used to evaluate the liability of the triplicate data in all participants; subjects with intraclass correlation coefficients <0.7 were excluded. All participants were instructed to remain quiet and still during the entire duration of the measurements.

### 2.4. Hearing Assessment

Otoscopic examination and hearing tests were performed on the day before WBT in all participants. Hearing level was determined using auditory steady-state response, auditory brainstem response, conditioned orientated response, or pure tone audiogram depending on the subject’s ability. Hearing level was defined as the average of the hearing threshold at 0.5, 1.0, and 2.0 kHz.

### 2.5. Statistics

WBA under peak pressure and RF were compared between the two groups. The analysis plans were pre-registered and involved primary outcomes related to RF and absorbance, with predetermined thresholds of clinical significance. The Shapiro–Wilk test was used to check the normality of WBA values at each frequency from 226 to 8000 Hz and RF. Since the data were not normally distributed (*p* < 0.05), the Wilcoxon rank sum test was used to compare the WBA and RF values between groups. Receiver operating characteristic (ROC) analysis was used to determine the usefulness of WBT for diagnosing EVA. Statistical analyses were performed using IBM SPSS Statistics version 28.0.1.0 (IBM Corp., Armonk, NY, USA), with *p* < 0.05 considered statistically significant.

## 3. Results

WBT was used to evaluate a total of 79 ears: 40 ears in 25 patients (19 male ears, 20 female ears) in the EVA group and 39 ears in 28 subjects (17 male ears, 22 female ears) in the control group. The median age at WBT tended to be higher in the EVA group than in the control group (14 [4–49] vs. 12 [4–48] years; Mann–Whitney, U = 754.000; z = −0.256; *p* = 0.798). The demographic data are presented in Table 2.

### 3.1. Comparison of Absorbance

**Figure 2:**
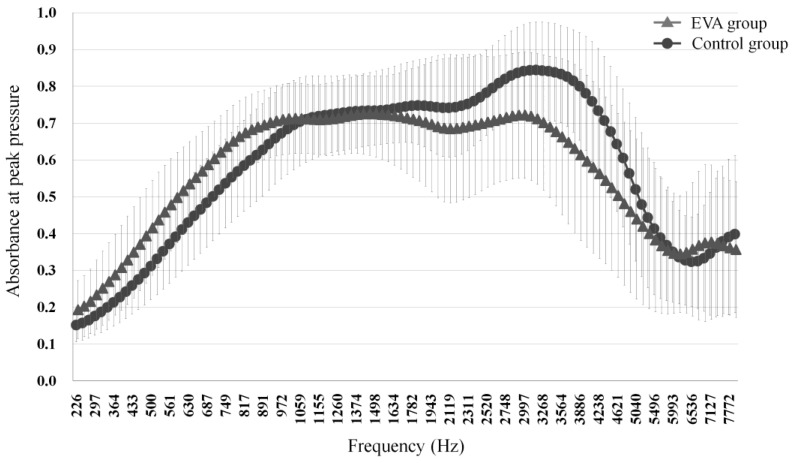
Mean absorbance curves at peak pressure against frequency group. The error bars represent ±1 standard deviation from the mean.

The mean absorbance under peak pressure of the EVA group was significantly higher at frequencies around 226–917 Hz (*p* < 0.05, Mann–Whitney U) and significantly lower at frequencies around 2520–4896 Hz (*p* < 0.05, Mann–Whitney U) compared to the control group (Figure 2).

### 3.2. Comparison of RF

**Figure 3:**
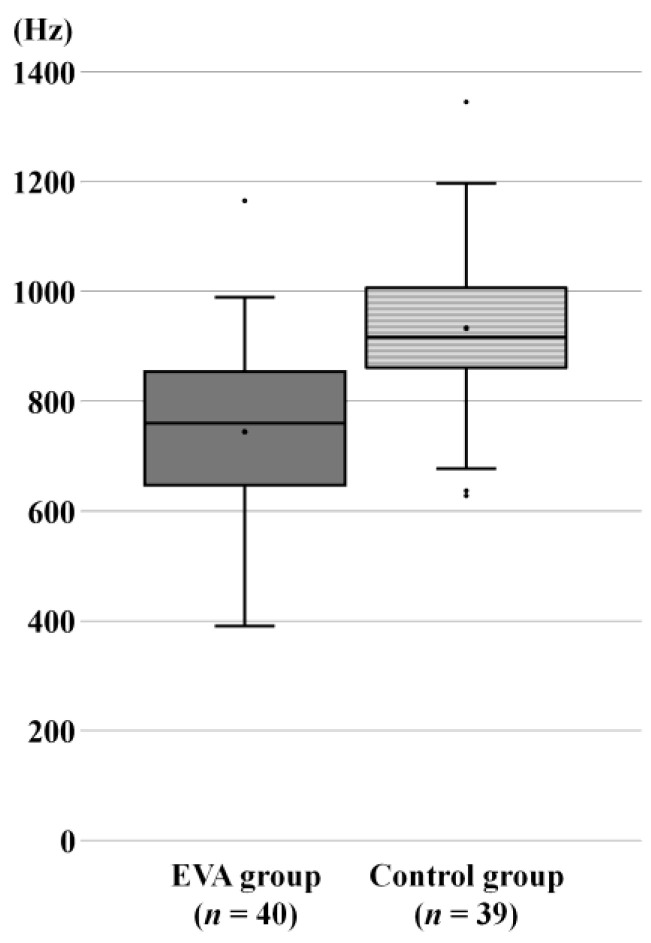
Comparison of RF between the control group and EVA group. The mean RF was significantly lower in the EVA group than in the control group.

The mean RF was 751 (391–1165) Hz and 933 (628–1346) Hz in the EVA and control groups, respectively. The mean RF in patients with EVA was significantly lower than that in controls (U = 296.500; z = −4.741; *p* < 0.001; Mann–Whitney U) (Figure 3).

### 3.3. ROC Curve Analysis for Absorbance and RF

**Figure 4:**
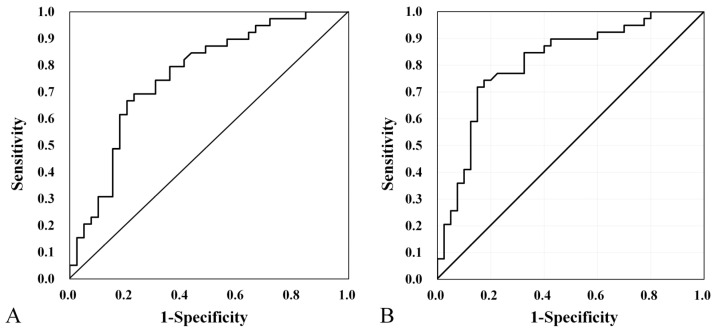
Receiver operating characteristic (ROC) curve analysis for absorbance at 3776 Hz (**A**). Area under the curve, 0.771 (95% confidence interval, 0.670–0.871). ROC analysis for RF in control and EVA groups (**B**). AUC, 0.801 (95% confidence interval, 0.712–0.907).

ROC curve analysis was used to evaluate the usefulness of absorbance and RF in diagnosing EVA. Among the frequencies at which significant differences in absorbance were observed (i.e., 226–917 Hz and 2520–4896 Hz), the most significant difference (*p* < 0.001) was found in the ranges of 408–794 Hz and 2997–4362 Hz. Thus, using ROC curve analysis, we evaluated the ability of WBT to discriminate between EVA patients and controls in the frequency ranges of 226–917 Hz and 2520–4896 Hz. The ROC curve analysis yielded a maximum area under the curve (AUC) of 0.771 (95% confidence interval, 0.670–0.871) (Figure 4A). The maximum sum of specificity and sensitivity was observed at 3776 Hz, with an absorbance of 0.782. WBT had a sensitivity, specificity, positive predictive value, and negative predictive value of 69.2%, 77.8%, 77.5%, and 69.2%, respectively, for detecting EVA.

ROC curve analysis was used to evaluate RF, yielding an AUC of 0.810 (95% confidence interval, 0.712–0.907). The maximum sum of specificity and sensitivity was observed at an RF of 888 Hz (Figure 4B). RF had a sensitivity, specificity, positive predictive value, and negative predictive value of 74.4%, 82.5%, 76.7%, and 80.6%, respectively, for detecting EVA.

### 3.4. Effect of Age on Diagnostic Accuracy

To investigate the effect of age on diagnostic accuracy, we examined the diagnostic accuracy of EVA using RF in participants before puberty (age ≤ 15 years, child group) and after puberty (age ≥ 16 years, adult group). This was performed because development of the temporal bone pneumatization typically concludes by the age of puberty [22,23].

**Figure 5:**
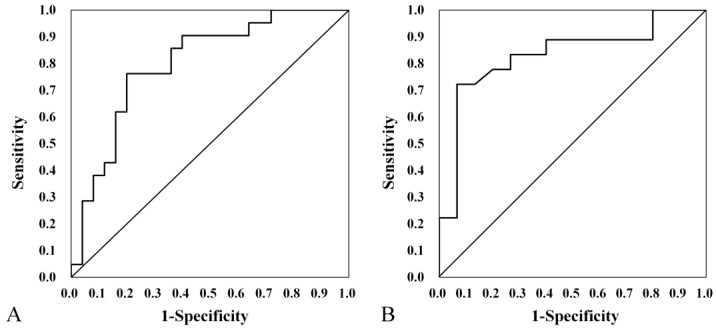
Receiver operating characteristic (ROC) curve analysis for RF in the (**A**) child group (area under the curve [AUC], 0.800 [95% confidence interval, 0.684–0.979]) and (**B**) adult group (AUC, 0.831 [95% confidence interval, 0.684–0.979]).

In the child group (25 patients with EVA and 21 controls), the ROC curve analysis yielded a maximum AUC of 0.800 (95% confidence interval, 0.670–0.936). The maximum sum of specificity and sensitivity was observed at an RF of 888 Hz (Figure 5A). RF had a sensitivity, specificity, positive predictive value, and negative predictive value of 80.0%, 76.2%, 80.0%, and 76.2%, respectively, for detecting EVA.

In the adult group (15 patients with EVA and 18 controls), the ROC curve analysis yielded a maximum AUC of 0.831 (95% confidence interval, 0.684–0.979). The maximum sum of specificity and sensitivity was observed at an RF of 891.67 Hz (Figure 5B). RF had a sensitivity, specificity, positive predictive value, and negative predictive value of 93.3%, 72.2%, 83.7%, and 92.3%, respectively, for detecting EVA.

### 3.5. Correlation Between EVA Size and RF

**Figure 6:**
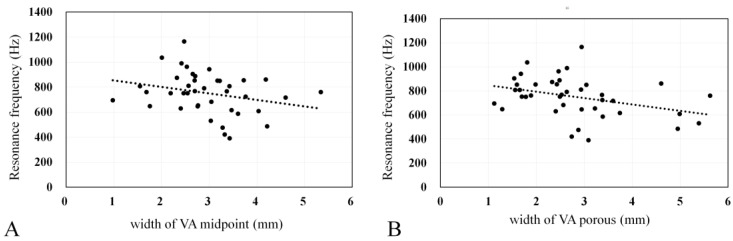
Correlations of resonance frequency with (**A**) the VA midpoint width (r = −0.334); and (**B**) the VA porous width (r = −0.402). The line represents the best-fit regression line.

The mean VA midpoint and porous widths were 2.96 and 2.79 mm, respectively, in the EVA group. Both VA midpoint and porous widths demonstrated good interobserver agreement (interobserver variability, 0.926 and 0.921, respectively), with higher agreement in the former. In the EVA group, significant correlations were found between the VA midpoint width and RF (Spearman; *p* = 0.035; r = −0.334), as well as between the VA porous width and RF (Spearman; *p* = 0.01; r = −0.402) (Figure 6).

## 4. Discussion

EVA is an auditory–vestibular disorder caused by the abnormal exposure of the inner ear to the surrounding structures, known as third window [24]. Normally, the inner ear cavity, which is surrounded by the bones of the otic capsule and filled with fluid, is connected to surrounding anatomical structures such as the oval window and round window (i.e., the first and second windows, respectively). Meanwhile, other means of exposure of the inner ear to surrounding structures are known as third windows; these include the cochlear aqueduct, which connects the scala tympani of the cochlea with the posterior fossa, and VA, which connects the vestibule with the posterior fossa [25]. Normally, third windows are anatomically thin tubes that have high impedance and block sound flow; thus, third windows are thought to have a little effect on hearing [26]. Conversely, EVA and SSCD act as a pathological third window, which exposes the inner ear to the surrounding structures, thus affecting sound transmission in the inner ear and causing hearing loss [24]. Hearing loss due to the presence of a third window often presents as mixed hearing loss, with an air–bone gap in low frequencies and severe high-frequency hearing loss that is fluctuating and progressive [27]. Nevertheless, the exact mechanism of conductive hearing loss in EVA patients remains unclear. Third windows such as EVA could possibly shunt acoustic energy outside the cochlea, redirecting it away from the inner ear and thus reducing sound pressure. The third window also reduces impedance in the scala vestibuli, which increases the pressure difference between the scala vestibuli and scala tympani on either side of the basilar membrane of the cochlea, improving bone conduction. This is in line with previous studies, which state that many patients with third window suffer from conductive hearing loss due to an increase in the bone conduction threshold [27,28]. Thus, regarding the effect of EVA on sound conduction, it is necessary to consider the following: (1) increased stiffness because of the transmission of cerebrospinal fluid pressure to the inner ear via EVA, leading to increased inner ear pressure and restricted mobility of the stapes, and (2) decreased impedance due to increased mass of VA and the endolymphatic sac.

Third window is thought to affect the absorbance of acoustic energy. In SSCD, which has a third window like EVA, increased absorbance has been reported at low frequencies [29,30]. Similarly, this study identified increased absorbance in the EVA group at low frequencies (226–917 Hz) compared to controls (Figure 2). This is thought to be related to the decrease in impedance due to the increased mass of VA and the endolymphatic sac. Similar results have been reported in patients with endolymphatic hydrops, with absorbance at low frequencies (560–600 Hz) being higher in ears with severe endolymphatic hydrops versus those with mild or no endolymphatic hydrops [31]. It is thought that the presence of EVA creates a sound energy shunt from the inner ear to the posterior fossa, and that the increased volume of the endolymphatic sac and duct [32] reduces inner ear impedance, resulting in increased absorption at a low frequency range [33].

The EVA group had significantly lower absorbance than controls at middle–high frequencies around 2520–4896 Hz, as indicated by the frequency–absorption curve at peak pressure (Figure 2). Stiffness and mass are factors that affect sound transmission in the middle ear. Stiffness has a significant effect on the transmission efficiency of low-frequency sound, whereas mass has a significant effect on the transmission efficiency of high-frequency sound [15]. High-frequency sound has a short wavelength, and an increased inner ear volume caused by EVA leads to a decrease in the transmission of high-frequency sound [34]. This could explain the lower absorbance of EVA at a high frequency range versus the control group.

Similar to absorbance, RF is also determined by mass and stiffness in the middle ear and inner ear. Mass and stiffness are determined by several factors: the middle ear sound conductive system, the volume of the middle ear, air pressure, the tonus of the middle ear muscles, and the mechanical immittance of the cochlea [33]. An increase in RF can be caused by a decrease in mass or an increase in stiffness, and vice versa. No abnormal findings of the middle ear or ossicles have been reported among EVA patients, except for EVA-associated with other syndromic causes of hearing loss such as branchio-oto-renal syndrome and Waardenburg syndrome [13]. In this report, patients with abnormal findings on tympanic membrane examination, tympanometry using 226 Hz, and CT imaging were excluded, and thus the changes in ABS and RF observed in EVA patients are likely influenced by EVA-associated changes in mass and stiffness. As an effect of EVA, abnormally high perilymph pulsation is often observed clinically in cases of cochlear implant surgery among patients with EVA [12,15,35], which suggests an increased inner ear pressure due to the continuous transmission of cerebrospinal fluid pressure to the cochlea via EVA. From the perspective of middle ear impedance, the increase in inner ear pressure restricts the movement of the stapes [6], and the increasing stiffness causes RF to shift to higher frequencies [33,36]. From the perspective of cochlear impedance, the presence of EVA reduces cochlear impedance by forming a sound energy shunt from the cochlea to the posterior fossa and increasing the volume of the endolymphatic sac and duct [32,33]. Therefore, the larger the VA size, the greater the decrease in RF [33]. The EVA group had lower RF than the control group (Figure 3); this is likely because the decrease in cochlear impedance due to increased mass had a greater effect versus the increase in ossicular stiffness due to increased inner ear pressure.

The correlations between VA width and RF were examined (Figure 6). The correlation coefficient between VA midpoint width and RF was −0.334, while that between VA porous width and RF was −0.402; these are considered weak. In this report, the diameter of VA, rather than its volume, was used to indicate the degree of enlargement because it is difficult to distinguish between the enlarged endolymphatic sac, the surrounding cerebrospinal fluid, and blood vessels on CT. As a result, the degree of vestibular aqueduct enlargement could not be evaluated accurately, which can explain the weak correlation between VA size and RF.

ROC analysis was used to determine the usefulness of absorbance and RF in diagnosing EVA (Figure 4), yielding AUC values of 0.782 and 0.801, respectively. Absorbance at 3776 Hz and RF both had moderate or higher diagnostic values, suggesting that RF may be more useful for diagnosis than absorbance. While inner ear malformations account for approximately 20% of all congenital hearing loss, which is often detected in infancy [35,37], EVA is the most common inner ear malformation associated with hearing loss [38,39]. However, some inner ear malformations such as EVA may be missed on newborn hearing screening, leading to a delayed diagnosis [40]. Consequently, children with progressive forms of hearing loss remain undiagnosed during their critical period of speech development and neuroplasticity [41]. Current diagnostic approaches heavily rely on CT and MRI imaging and genetic-related factors, which, in turn, bring a number of challenges with respect to radiation exposure in a pediatric population, such as high cost and accessibility in several health settings. These limitations often result in diagnostic delays with potential negative implications for treatment outcomes. Although the genetics of HL has evolved in recent years, there is currently limited knowledge in this area [41]. In contrast, WBT only requires a small amount of additional training for audiologists before being used as a screening tool; this can potentially save time, effort, and cost compared with direct imaging. In addition, there was consistent diagnostic accuracy between the child and adult groups, although it was slightly higher in the adult group in our study (Figure 5). Although the standardization of measurement protocols and clear referral pathways for clinical application need to be considered, our results would suggest that WBT might represent a clinically useful screening test for EVA, particularly in settings where imaging is not readily available. The negative predictive value in our study is 80.6%, suggesting that WBT may be more useful in ruling out EVA, thereby potentially reducing unnecessary imaging studies.

Although these preliminary results regarding diagnostic performance are promising, several limitations need to be considered. First, the nature of our two-center study may limit generalizability. Second, although the control group was age- and sex-matched, it might not fully represent the spectrum of patients that would most commonly require screening for EVA. Third, collecting population-based normative data is crucial for the standardization of WBA because its patterns are influenced by ethnicity, gender, and age [42]. To address this, we matched subjects by ethnicity, gender, and age between both groups; nevertheless, these results are only directly applicable to Japanese populations.

## 5. Conclusions

Compared to controls, patients with EVA had higher absorbance in the low frequency range and lower absorbance in the high frequency range, which is consistent with previous reports. The optimal index for diagnosing EVA using WBT was RF, with an AUC of 0.801 on ROC analysis. RF also had a sensitivity, specificity, positive predictive value, and negative predictive value of 74.4%, 82.5%, 76.7%, and 80.6%, respectively. These results suggest that WBT may be useful for diagnosing EVA.

## Figures and Tables

**Table 1:** Inclusion criteria.

Control group	
1	No history of acquired ear diseases
2	No acute or chronic upper respiratory inflammation
3	Normal findings on pretest otoscopic examination
4	Type-A tympanometry
5	No acute or chronic upper respiratory disease
6	Normal hearing level (≤25 dBHL from 500 to 2000 Hz, air-bone gap ≤10 dB from 500 to 2000 Hz)
Enlarged vestibular aqueduct (EVA) group	
1	Patients who meet criteria 1–5 in the control group
2	VA with a diameter of >1.5 mm at the midpoint
3	No history of middle or inner ear surgery

**Table 2:** Demographic data.

	EVA Group	Control Group	*p*-Value
Number of subjects	25	28	
Number of ears	40	39	
Sex			
Male	21 (52.5%)	23 (59%)	0.361
Female	19 (47.5%)	16 (41%)	
Side of WBT			
Right	19 (48%)	21 (54%)	0.651
Left	21 (52%)	18 (46%)	
Age at WBT (years)	14 (4–49)	12 (4–48)	0.822
Hearing level (dB)	80 ± 22.2	9.0 ± 4.0	<0.001

WBT: wideband tympanometry.

## Data Availability

All collected and analyzed data in this study are presented in this published article.
